# Chronic consumption of energy drinks and the risk of developing metabolic syndrome in rats

**DOI:** 10.7717/peerj.20926

**Published:** 2026-03-10

**Authors:** Ahlam Saleh Alhajri

**Affiliations:** Food Science and Nutrition Department, College of Agricultural and food Sciences, King Faisal University, Al-Ahsa, Eastern Province, Saudi Arabia

**Keywords:** Energy drinks, Caffeine, High dose, Biochemical alterations, Histopathological changes, Liver toxicity

## Abstract

The consumption of energy drinks (EDs) has notably increased, particularly among adolescents and young adults, due to their perceived benefits in enhancing physical and cognitive performance. However, growing evidence points to their potential adverse health effects, raising concerns regarding their safety. This study aimed to evaluate and compare the metabolic impacts of three commercially available EDs on male albino rats. A total of 42 rats were randomly assigned into seven groups (*n* = 6 per group). The control group received a standard basal diet, while the remaining groups administered the three EDs types (Red Bull, Power Horse, and Black) at doses of 10 or 20 mL/kg body weight, twice daily *via* oral gavage, over a period of eight weeks. At the end of the experiment, body weight gain, adiposity index, and multiple biochemical and physiological parameters were assessed. Serum analyses were performed to evaluate blood glucose levels, metabolic hormones (insulin and leptin), kidney function markers, liver enzymes, lipid profile, cardiovascular risk index, calcium levels, bone mineral density (BMD), and bone mineral concentration (BMC). The findings revealed that energy drink consumption, particularly at the higher dose (20 mL/kg bw) and with the third EDs type, induced significant adverse effects compared to controls. These included elevated blood glucose, leptin, liver enzymes, lipid profile, cardiovascular risk index, and serum calcium, alongside reduced insulin levels, High-density lipoprotein cholesterol (HDL-C), albumin, globulin, total protein, BMD, and BMC. Histopathological analysis of liver tissues showed evidence of cellular atrophy and structural damage. In conclusion, chronic intake of high doses of EDs may contribute to the development of metabolic disorders such as diabetes mellitus, obesity, cardiovascular risk, and osteoporosis. These findings underscore the need for public health awareness and regulation of EDs consumption, especially among vulnerable populations.

## Introduction

In recent years, energy drinks (EDs) have gained prominence as a distinct category of beverages, first introduced in the United States in 1997. EDs are non-alcoholic liquid beverages formulated with a combination of stimulants and energy-enhancing compounds designed to improve energy levels, stamina, athletic performance, and cognitive concentration (Costantino et al., 2023). These drinks have been widely adopted by various groups, including athletes and college students, who often perceive them as beneficial for health and performance (Bedi, Dewan & Gupta, 2014).

Caffeine remains the principal active component in EDs, although many formulations include additional dietary supplements. As a methylxanthine, caffeine is widely recognized for its stimulatory effects on the central nervous system (CNS), leading to increased alertness, reduced fatigue, and improved cognitive performance (Breda et al., 2014). Caffeine content in EDs varies considerably, ranging from 50 to 505 mg per can or bottle, with concentrations between 2.5 and 171 mg per 28 mL, often exceeding the caffeine content of a typical 170 g cup of brewed coffee, which contains between 77 and 150 mg (Marinoni et al., 2022).

EDs also frequently contain a variety of other components, including natural plant extracts (*e.g.*, yerba mate, ginseng, guarana, ginkgo biloba, acai), macronutrients (proteins, carbohydrates), micronutrients (vitamins& minerals), and artificial sweeteners (such as sucralose and aspartame). Fruity variants may include juices from pineapple, apple, strawberry, carrot, and pomegranate ingredients often high in glucose (Nadeem et al., 2021). These products are commonly consumed before or after physical activity to boost energy levels and replace lost fluids and electrolytes, as they often contain sodium, potassium, and other hydration-promoting agents (Ariffin et al., 2022).

Energy drinks are commonly consumed for their stimulating effects; however, growing evidence highlights their potential contribution to cardiometabolic disorders. Excessive caffeine intake, particularly above ∼300 mg/day, has been associated with adverse cardiovascular outcomes such as elevated blood pressure and arrhythmias. Nevertheless, in the context of EDs and other bottled beverages, it is the high sugar content that has been more consistently implicated in the development of metabolic syndrome (MS). Frequent consumption of sugar-sweetened beverages has been linked to central obesity, insulin resistance, dyslipidemia, and hypertension, which together constitute the defining components of MS (Malik et al., 2010; Stanhope, 2016).

MS is a cluster of conditions including central obesity, dyslipidemia, hyperglycemia, hypertension, and endothelial dysfunction that collectively increase the risk of cardiovascular disease (CVD) and type 2 diabetes. It promotes a state of positive energy balance by enhancing insulin secretion in response to elevated dietary substrates, often driven by excessive caloric intake (Hamooya et al., 2025). This metabolic environment stimulates hepatic lipogenesis and the secretion of lipoprotein triglycerides. Simultaneously, insulin upregulates adipose tissue endothelial lipases, promoting fatty acid uptake and triglyceride storage in adipocytes. Chronic lipid accumulation induces the release of pro-inflammatory adipokines, which in turn impair insulin signaling, perpetuating hyperglycemia and chronic systemic inflammation (Graneri et al., 2021). Given the widespread and increasing consumption of EDs, the current experimental study aims to evaluate their biochemical impact using a controlled model involving normal rats.

## Material and Methods

### Animal experimental design

This study aimed to examine the biochemical effects of three types of EDs on healthy rats. Three popular ED brands Red Bull, Power Horse, and Black were used, all obtained from a local market in the Al-Ahsa Governorate, Saudi Arabia. The nutritional content of these EDs is presented in Table 1.

**Table 1:** The nutritional details of the tested energy drinks.

**Constitutes/100 ml**	**Type (1) Red Bull**	**Type (2) Black** **(lime of mint)**	**Type (3) Power Horse**
Energy	45.2 kcal	44 kcal	48 kcal
Protein	0.3 g	–	–
Fat	0.1 g	–	–
Carbohydrates	11.0 g	11.0 g	11.9 g
Taurin	0.4 g	0.4 g	0.4 g
Caffeine	32.0 mg	30.0 mg	32.0 mg
Niacin	8.0 mg	7.0 mg	8.0 mg
Vitamin B6	2.0 mg	1.4 mg	2.0 mg
Pantothenic acid	2.0 mg	1.98 mg	2.0 mg
Riboflavin	–	0.5 mg	0.06 mg
Vitamin B12	0.002 mg	–	2.0 mg
Sodium	38 g	0.1 g	–

The sample size was determined based on previous studies that investigated comparable physiological and biochemical parameters in rats. A total of 42 adult male Sprague Dawley albino rats (aged approximately three months and weighing 300 ± 5 g) were used. These animals were obtained from the animal facility at King Faisal University, Saudi Arabia. The rats were randomly allocated into seven groups, each comprising six animals, a design intended to provide sufficient statistical power while adhering to ethical guidelines and accounting for the availability of animals. Although a formal a priori sample size calculation was not performed, the selected group size (*n* = 6) aligns with standard practices in animal research and is generally considered adequate for detecting biologically significant differences with acceptable variability. All experimental procedures were reviewed and approved by the Research Ethics Committee, Deanship of Scientific Research, King Faisal University (Approval No. KFU-REC-2024-SEP-ETHICS2478).

In the laboratory, rats were housed in standard polycarbonate cages (40 × 25 × 20 cm) with corncob bedding. They were maintained under controlled environmental conditions: a 12-hour light/dark cycle, temperature of approximately 20–22 ^∘^C, and 40–60% relative humidity. Environmental enrichment, including cardboard tubes and wooden blocks, was provided to encourage natural behaviors and reduce stress. Rats were fed a standard laboratory chow and given water *ad libitum*. They were allowed a 1-week acclimatization period before the experimental procedures commenced.

Following the method of Reeves, Nielsen & Fahey Jr (1993), the animals were fed a basal diet composed of corn starch (69.5%), casein (10%), vitamin mixture (1%) (Campbell, 1963), salt mixture (4%), corn oil (10%) (Hegsted, Mills & Perkins, 1941), methionine (0.3%), bran (5%), and choline chloride (0.2%).

After acclimatization, the experimental animals were randomly allocated into seven groups (*n* = 6 per group). Group 1 served as the control and received only the basal diet. The remaining six groups were maintained on the basal diet and additionally administered EDs treatments for a period of eight weeks. Three commercial types of EDs were evaluated: Red Bull, Power Horse, and Black. Each ED was assessed at two dose levels (10 and 20 mL/kg body weight). Accordingly, six treatment groups were established: Group 2 and Group 3 received Red Bull at 10 and 20 mL/kg, respectively; Group 4 and Group 5 received Power Horse at 10 and 20 mL/kg, respectively; and Group 6 and Group 7 received Black at 10 and 20 mL/kg, respectively. The EDs were administered orally *via* gastric gavage twice daily, following the procedure described by Kassab & Tawfik (2018).

To minimize potential confounders, all rats were kept under identical conditions. Cage positions were rotated regularly to eliminate any cage-location effects. The order of measurements and sample collection was randomized to reduce potential measurement bias. Group allocations were known only to the researcher responsible for randomization; all other personnel involved in animal handling, outcome assessment, and data processing were blinded to group assignments.

Body weight and adiposity index were recorded. Blood and liver samples were collected for analysis. Fasting blood glucose, insulin, and leptin levels were assessed using standard enzymatic and immunoassay techniques. Markers of liver and kidney function, lipid profile, serum calcium, bone mineral concentration (BMC), and bone mineral density (BMD) were also measured using standard biochemical methods. Additionally, liver histopathology was examined using Hematoxylin and Eosin (H&E)-stained tissue sections.

### Inclusion and exclusion criteria

The study utilized male albino rats of the Sprague Dawley strain as the experimental model, selected based on predefined *a priori* inclusion criteria. All animals were 12 weeks of age and weighed approximately 130. ± 5 g. at the start of the study. A preliminary health screening was performed to confirm that the selected rats were free from any pre-existing metabolic disorders.

Exclusion criteria were also established prior to the study. Animals were excluded if they showed signs of illness or physiological abnormalities before the initiation of the trial, failed to adapt during the 7-day acclimatization period, or did not consume the minimum required amount of energy drink or standard diet during the intervention. It is noteworthy that no animals or data points were excluded from the final analysis.

#### Determination of body weight and adiposity index (AI)

Body weight was recorded weekly throughout the experimental period. Body weight gain was calculated using the equation described by Chapman, Castillo & Campbell (1959):

Body weight gain (g) = Final body weight (g)−Initial body weight (g).

The AI was determined according to the method of Francisqueti et al. (2017) by dividing the total fat mass, comprising epididymal, visceral, and retroperitoneal fat, by the final body weight: AI = (Epididymal fat + Visceral fat + Retroperitoneal fat)/Final body weight.

#### Blood sampling and liver organ

At the end of the experimental period, and following a 12-hour fast, the rats were anesthetized with urethane (1 g/kg, intraperitoneally; Sigma-Aldrich, Germany) (Van Asselt et al., 2017). Blood samples were then collected from the hepatic portal vein using sterile, dry centrifuge tubes (Strubbe, Bruggink & Steffens, 1998). The samples were allowed to clot for 30 min in a water bath maintained at 37 ^∘^C. Serum was subsequently separated by centrifugation at 3,000 rpm for 10 min. The resulting serum was aspirated into clean cuvette tubes and stored at –20 ^∘^C for later biochemical analysis (Saad et al., 2025).

Following sample collection, the rats were euthanized using carbon dioxide (CO_2_) inhalation, in accordance with the AVMA Guidelines for the Euthanasia of Animals (2020). CO_2_ was introduced at a controlled rate of 30–70% of the chamber volume per minute to induce unconsciousness and minimize distress (Underwood & Anthony, 2020). All procedures were conducted in compliance with institutional ethical standards and approved animal care protocols.

The livers were excised and rinsed with saline solutions to remove blood residues, and prepared for histological examination in accordance with the protocol of Drury & Wallington (1980).

#### Biochemical investigation

##### Blood glucose.

Blood glucose levels were measured using a commercial assay kit (Cat. No. 675, Riad Laboratory). Briefly, 10 µL of plasma was mixed with 1.0 mL of the working enzyme reagent, thoroughly vortexed, and incubated at 37 ^∘^C for 15 min. The resulting colorimetric reaction was quantified by measuring absorbance at 505 nm using a spectrophotometer. A blank sample containing distilled water was processed in parallel to calibrate the assay. A glucose standard was also included and treated under the same conditions. Glucose concentrations were calculated and expressed in mg/dL according to the procedure described by Frank, Shubha & D’Souza (2012).

##### Metabolic hormones.

Serum insulin levels were measured using a commercial enzyme-linked immunosorbent assay (ELISA) kit from Bio-Merieux (Lyon, France), following the method described by Huang & Czech (2013). Briefly, 25 µL of serum was added to microwells pre-coated with anti-insulin antibodies. Subsequently, 100 µL of enzyme conjugate was dispensed into each well, mixed for 5 s, and incubated at 25 ^∘^C for 30 min. The wells were then washed five times with a washing buffer. Afterward, 100 µL of solution A followed by 100 µL of solution B was added to each well and incubated for 15 min at room temperature. The reaction was stopped by adding 50 µL of 1 mol/L HCl to each well. Absorbance was read at 450 nm using a microplate reader. Insulin concentrations were expressed as ng/ML.

Leptin levels were quantified using a leptin-specific ELISA kit (DRG Diagnostics, Marburg, Germany), according to the manufacturer’s instructions, as referenced by Acar et al. (2018).

Thyroid hormone markers, including total triiodothyronine (T3), total thyroxine (T4), and thyroid-stimulating hormone (TSH), were measured using *in vitro* diagnostic radioimmunoassay techniques with the Immunolite 2000 analyzer, as described by El-Wakf, Hassan & Elsaid (2009). Free triiodothyronine (FT3) and free thyroxine (FT4) levels were also assessed using competitive radioimmunoassay, based on the methods of Henning et al. (2014) and Liu et al. (2025).

##### Liver functions.

Total protein and albumin concentrations were measured using commercial kits (Human-GmbH, Germany). Albumin determination was based on the bromocresol green (BCG) binding assay, where 10 µL of blood plasma was mixed with the reagent buffer. After 3 min of incubation, absorbance was measured at 546 nm, as described by Ettinger & Feldman (2005) and Kaneko, Harvey & Bruss (2008). Globulin levels were calculated by subtracting the albumin concentration from the total protein concentration, according to the method of Busher (1990).

Serum enzyme activities, including aspartate transaminase (AST), alanine transaminase (ALT), and alkaline phosphatase (ALP), were determined using colorimetric methods with absorbance measured at 340 nm. The assays employed 2,4-dinitrophenylhydrazine as a substrate, following the protocols outlined by Tietz (1995), Young & Friedman (2001), and Rifai (2017).

##### Kidney functions.

Serum urea, uric acid, and creatinine concentrations were determined using enzymatic colorimetric assays. The intensity of the developed color, proportional to the concentration of each analyte, was measured spectrophotometrically at 600 nm, following the protocols outlined by Young & Friedman (2001) and Rifai (2017).

##### Lipid profile.

Total cholesterol (TC) was measured using the enzymatic endpoint CHOD-PAP method as described by Robinet et al. (2010), while triglycerides (TG) were determined by the enzymatic glycerol phosphate oxidase–peroxidase method (Miller et al., 2011). High-density lipoprotein cholesterol (HDL-C), low-density lipoprotein cholesterol (LDL-C), and very-low-density lipoprotein cholesterol (VLDL-C) were assessed using a homogeneous enzymatic direct assay (Matsushima-Nagata et al., 2021). The atherogenic index (AI), coronary risk index (CRI), and cardiac risk ratio (CRR) were calculated using the equations of Dobiasova (2004), Kikuchi Hayakawa et al. (1998), and Bhardwaj et al. (2013), respectively. Specifically, AI was calculated as log (TG/HDL-C), CRR as LDL-C/HDL-C. and CRI as TC/HDL-C.

##### Serum calcium, BMC and BMD.

Total BMD was measured using dual-energy X-ray absorptiometry (DXA) scans obtained from a QDR-4500A fan-beam densitometer and performed by certified radiologic technologists, as described by Hitz, Jensen & Eskildsen (2007). Serum calcium levels were determined using the Beckman Synchron LX20 analyzer (Kaiyu et al., 2021). BMC of the whole body was assessed with a Hologic QDR-1000 W DXA scanner (Hologic Inc., Waltham, MA, USA), according to the method of Abrahamsen et al. (1995).

#### Histopathological investigation of the liver

Small liver specimens were collected from the experimental groups and immediately fixed in neutral buffered formalin. The tissues were subsequently dehydrated through graded ethanol concentrations (70%, 80%, and 90%), cleared in xylene, and embedded in paraffin wax. Sections of 4–6 µm thickness were cut and stained with Hematoxylin and Eosin, following the protocol described by Bancroft & Gamble (2008).

#### Statistical analysis

Data were analyzed using one-way analysis of variance (ANOVA) followed by Tukey’s *post-hoc* test to compare differences among groups. Results are expressed as mean ± standard deviation (SD), and a *p*-value of less than 0.05 was considered statistically significant. Statistical analyses were performed using SPSS version 27 (IBM Corp., Armonk, NY, USA).

## Results

### Body weight gain and adiposity index

After eight weeks of administration of different EDs at doses of 10 and 20 mL/kg body weight, the control group showed significantly lower body weight gain compared to all treated groups. The group receiving 20 mL/kg of the third type of EDs exhibited the highest increase in body weight gain, recording a 40.79% rise compared to the control. Groups 5 and 6 showed no significant difference between each other, with increases of 21.36% and 23.11%, respectively, relative to the control (Table 2). Regarding the adiposity index, group 7 presented the highest mean value, which was statistically significant (*P* ≤ 0.05). No significant differences in adiposity index were observed between groups 3 and 6 or between groups 2 and 4.

**Table 2:** The mean and standard deviation of body weight gain and adiposity index of normal albino rats.

Groups	BWG (g/8 weeks)	Exchange from control group (%)	Adiposity index
Control group (G1)	39.89 ± 0.91^f^	—	0.34 ± 0.03^e^
10% ED1 (G2)	45.11 ± 0.23^d^	13.09	0.43 ± 0.09^d^
20% ED1 (G3)	51.05 ± 0.32^b^	27.98	0.56 ± 0.15^b^
10% ED2 (G4)	43.21 ± 1.05^e^	8.32	0.40 ± 0.05^d^
20% ED2 (G5)	48.41 ± 1.01^c^	21.36	0.52 ± 0.03^c^
10% ED3 (G6)	49.11 ± 0.76^c^	23.11	0.62 ± 0.18^b^
20% ED3 (G7)	56.16 ± 0.08^a^	40.79	0.88 ± 0.03b^a^
(LSD)	1.06		0.08

**Notes.**

Values are mean ± SD. Values in the same column showing the same superscript letters are not statistically significantly different at (*p* ≤ 0.05).

### Effects on blood glucose, insulin, and leptin hormones

As shown in Table 3, fasting blood glucose, insulin, and leptin hormone levels were significantly affected by EDs consumption. All EDs groups exhibited statistically significant increases in blood glucose and leptin hormone levels, alongside a significant decrease in insulin hormone compared to controls. The third EDs type produced the most pronounced effects, with the highest mean blood glucose and leptin hormone and the lowest insulin hormone levels. Long-term high-dose administration (20 mL/kg) of the third EDs (group 7) intensified these adverse effects, with blood glucose and leptin increasing by 102.97% and 43.36%, respectively, while insulin decreased by 48.68% relative to controls.

**Table 3:** Mean values and standard deviation of blood glucose, insulin hormone and leptin hormone of normal albino rats.

Groups	Blood glucose (mg/dl)	Insulin hormone (ng/ml)	Leptin hormone (pg/ml)
Control group (G1)	101.76 ± 3.53^g^	3.78 ± 0.031^a^	434.92 ± 8.03^g^
10% ED1 (G2)	127.78 ± 5.01^e^	3.35 ± 0.053^c^	481.76 ± 5.74^e^
20% ED1 (G3)	187.65 ± 4.71^b^	2.11 ± 0.017^f^	569.34 ± 3.99^b^
10% ED2 (G4)	111.54 ± 3.39^f^	3.52 ± 0.08^b^	442.64 ± 2.06^f^
20% ED2 (G5)	170.31 ± 1.23^c^	2.33 ± 0.09^e^	543.75 ± 4.92^c^
10% ED3 (G6)	141.89 ± 3.86^d^	3.12 ± 0.011^d^	507.98 ± 6.37^d^
20% ED3 (G7)	206.55 ± 2.34^a^	1.94 ± 0.07^g^	623.54 ± 5.87^a^
LSD	8.68	0.15	9.67

**Notes.**

Values are mean ± SD. Values in the same column showing the same superscript letters are not statistically significantly different at (*p* ≤ 0.05).

### Serum total protein, albumin, globulin, and Alb/Glb ratio

Data presented in Table 4 demonstrate that the control group had significantly higher mean serum total protein, albumin (Alb), globulin (Glb), and Alb/Glb ratio than the EDs-treated groups (*P* ≤ 0.05). The third EDs group showed the most significant reductions across all parameters (*P* ≤ 0.05). The higher dose (20 mL/kg) exacerbated these effects more than the lower dose. Albumin was the most affected parameter across all tested doses. Groups 2 and 4 showed no significant differences except in the Alb/Glb ratio.

**Table 4:** Mean values and standard deviation of blood glucose, insulin hormone and leptin hormone of normal albino rats.

Groups	T.P (mg/dl)	Alb (mg/dl)	Glb (mg/dl)	Alb/Glb ratio
Control group (G1)	10.38^a^± 0.13	4.97^a^± 0.001	5.41^a^± 0.007	0.92^a^± 0.019
10% ED1 (G2)	8.58^b^± 0.06	3.22^b^± 0.21	5.36^b^± 0.016	0.60^c^± 0.026
20% ED1 (G3)	5.82^d^± 0.45	1.91^d^± 0.09	3.91^e^± 0.02	0.49^e^± 0.021
10% ED2 (G4)	9.30^b^± 0.29	3.98^b^± 0.12	5.32^b^± 0.008	0.75^b^± 0.024
20% ED2 (G5)	6.19^d^± 0.16	2.13^c^± 0.03	4.06^d^± 0.047	0.52^d^± 0.036
10% ED3 (G6)	8.12^c^± 0.37	2.89^c^± 0.006	5.23^c^± 0.031	0.55^d^± 0.017
20% ED3 (G7)	4.82^e^± 0.28	1.45^d^± 0.04	3.37^f^± 0.051	0.43^f^± 0.018
L.S.D	0.75	0.76	0.07	0.04

**Notes.**

Values mean ± SD. Values in the same column sharing the same superscript letters are not statistically significantly different (*p* ≤ 0.05).

### Kidney function parameters

Table 5 illustrates the impact of EDs consumption on serum uric acid, urea, and creatinine levels. Kidney function markers were dose- and EDs type-dependent, with the highest levels observed in the group consuming 20 mL/kg of the third EDs, followed by the same dose of the first EDs, both significantly elevated compared to controls. No significant differences were noted between groups consuming 10 mL/kg of types 2 and 3. Urea and uric acid appeared more sensitive to EDs exposure than creatinine.

**Table 5:** Mean values and standard deviation of the kidney functions of normal albino rats.

Groups	Uric acid (mg/dl)	Urea (mg/dl)	Creatinine (mg/dl)
Control group (G1)	2.28 ± 0.005^f^	16.63 ± 1.89^f^	0.82 ± 0.007^f^
10% ED1 (G2)	3.24 ± 0.19^d^	24.79 ± 1.64^d^	1.13 ± 0.043^d^
20% ED1 (G3)	5.82 ± 0.004^b^	31.43 ± 1.84^b^	1.60 ± 0.008^b^
10% ED2 (G4)	2.56 ± 0.17^e^	19.78 ± 1.53^e^	0.98 ± 0.11^e^
20% ED2 (G5)	4.41 ± 0.02^c^	27.54 ± 0.81^c^	1.32 ± 0.06^c^
10% ED3 (G6)	3.19 ± 0.018^d^	24.83 ± 0.006^d^	1.16 ± 0.01^d^
20% ED3 (G7)	6.09 ± 0.21^a^	35.04 ± 1.45^a^	1.76 ± 0.03^a^
LSD	0.25	2.15	0.14

**Notes.**

Values are mean ± SD. Values in the same column sharing the same superscript letters are not statistically significantly different at (*p* ≤ 0.05).

### Liver enzymes

As indicated in Table 6, serum AST, ALT, and ALP levels were elevated in all EDs groups compared to controls. Enzyme levels were significantly higher (*P* ≤ 0.05) in groups receiving 20 mL/kg than in those receiving 10 mL/kg. The third EDs type elicited the greatest increase in these liver enzymes.

**Table 6:** Mean values and standard deviation of liver enzymes of normal albino rats.

Groups	AST (U/L)	ALT (U/L)	ALP (U/L)
Control group (G1)	29.86 ± 1.77^f^	30.31 ± 2.01^f^	77.73 ± 1.72^f^
10% ED1 (G2)	40.77 ± 0.92^d^	39.79 ± 1.76^d^	97.73 ± 4.23^d^
20% ED1 (G3)	51.42 ± 1.42^b^	49.91 ± 0.008^b^	118.73 ± 1.06^b^
10% ED2 (G4)	37.13 ± 2.42^e^	35.51 ± 1.28^e^	89.19 ± 1.56^e^
20% ED2 (G5)	46.22 ± 1.22^c^	44.95 ± 1.25^c^	106.23 ± 3.25^c^
10% ED3 (G6)	43.82 ± 3.06^d^	40.11 ± 0.26^d^	98.43 ± 1.13^d^
20% ED3 (G7)	55.11 ± 2.01^a^	52.92 ± 1.05^a^	122.32 ± 2.35^a^
LSD	3.11	2.07	3.53

**Notes.**

Values are mean ± SD. Values in the same column sharing the same superscript letters are not statistically significantly different at (*p* ≤ 0.05).

### Lipid profile and cardiovascular risk

**Figure 1:**
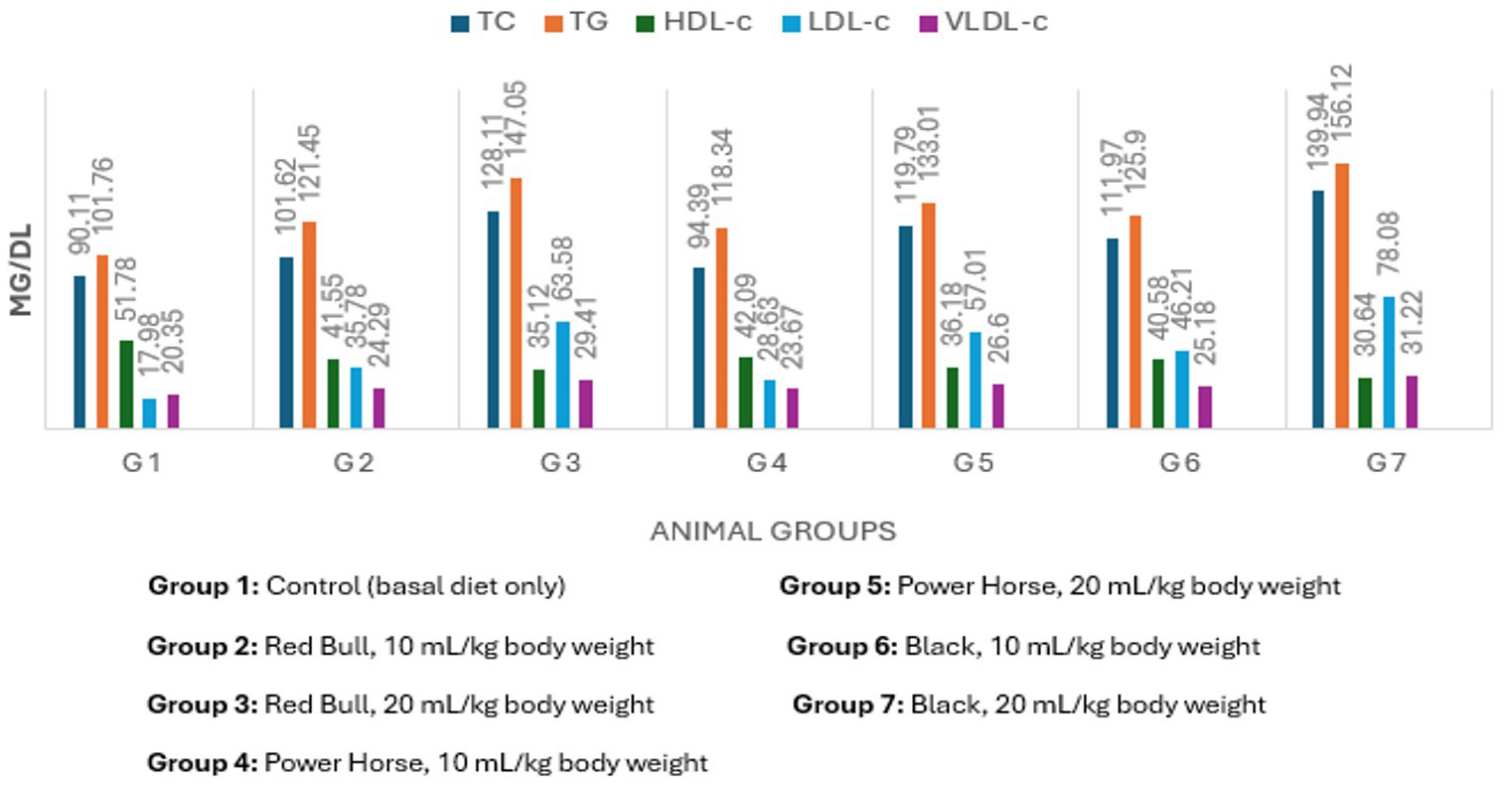
Mean values of lipid fractions of normal albino rats (mgdl). Group 1: Control (basal diet only) Group 2: Red Bull, 10 mL/kg body weight Group 3: Red Bull, 20 mL/kg body weight Group 4: Power Horse, 10 mL/kg body weight Group 5: Power Horse, 20 mL/kg body weight Group 6: Black, 10 mL/kg body weight Group 7: Black, 20 mL/kg body weight.

**Figure 2:**
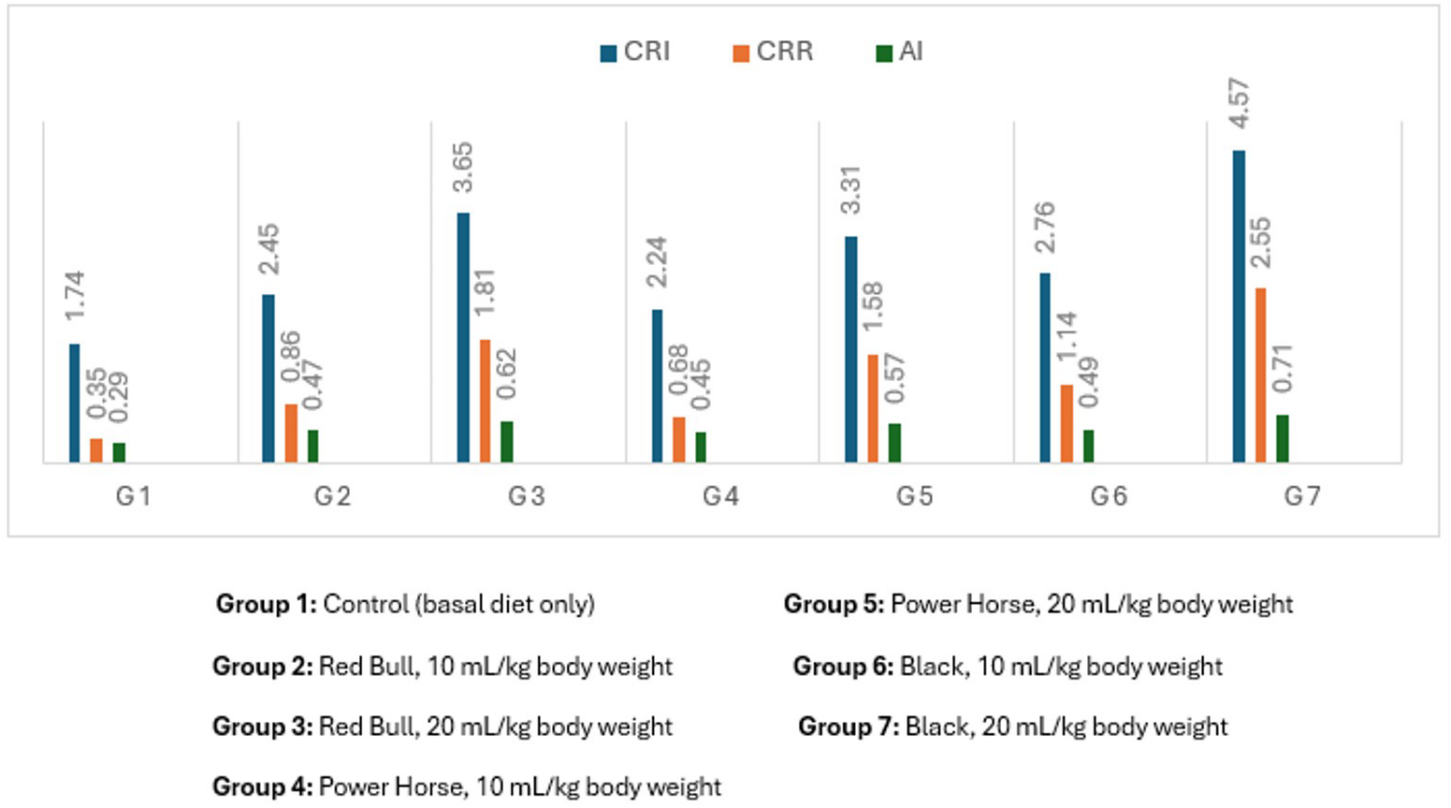
Mean values of cardiovascular risk index of normal albino rats. Group 1: Control (basal diet only) Group 2: Red Bull, 10 mL/kg body weight Group 3: Red Bull, 20 mL/kg body weight Group 4: Power Horse, 10 mL/kg body weight Group 5: Power Horse, 20 mL/kg body weight Group 6: Black, 10 mL/kg body weight Group 7: Black, 20 mL/kg body weight.

Figure 1 shows that all EDs-treated groups had significantly altered lipid profiles compared to controls. The highest lipid profile values occurred in groups receiving 20 mL/kg doses, while HDL-c decreased as the EDs dose increased. The third EDs type caused the most significant lipid profile disruption. Figure 2 illustrates that cardiovascular risk indices (CRI and AI) were elevated in all treated groups relative to controls, with the highest value observed in the high-dose third EDs group.

### Serum calcium, bone mineral density, and bone mineral concentration

Table 7 reveals serum calcium concentrations ranging from 8.11 mg/dL in controls to 14.21 mg/dL in group 7 (20 mL/kg of the third EDs). Control rats exhibited the highest BMD and BMC at 0.151 ± 0.003 g/cm^2^ and 0.169 ± 0.0004 g, respectively. All EDs-treated groups showed significantly reduced BMD and BMC compared to controls. Higher EDs doses were associated with increased serum calcium and reduced BMD and BMC, with the third EDs type causing the most pronounced negative effects, followed by the first.

**Table 7:** Mean values and standard deviation of serum calcium, bone mineral concentration (BMC) and bone mineral density (BMD) of normal albino rats.

Groups	Serum calcium (mg/dl)	BMD (g/cm^2^)	BMC (g)
Control group (G1)	8.11^e^± 0.26	0.151^a^ ± 0.007	0.169^a^ ± 0.001
10% ED1 (G2)	9.91^c^± 0.90	0.133^c^ ± 0.003	0.140^c^ ± 0.005
20% ED1 (G3)	13.46^a^ ± 0.53	0.112^e^± 0.001	0.116^e^ ± 0.006
10% ED2 (G4)	9.17^d^ ± 0.67	0.141^b^ ± 0.004	0.158^b^ ± 0.004
20% ED2 (G5)	11.94^b^ ± 1.01	0.122^d^ ± 0.001	0.127^d^ ± 0.005
10% ED3 (G6)	10.27^c^± 0.008	0.128^c^ ± 0.003	0.131^c^ ± 0.007
20% ED3 (G7)	14.21^a^ ± 0.16	0.098^e^ ± 0.005	0.115^e^ ± 0.005
LSD	1.03	0.007	0.009

**Notes.**

Values are mean ± SD. Values in the same column sharing the same superscript letters are not statistically significantly different at (*p* ≤ 0.05).

### Thyroid function

Table 8 shows significant differences in thyroid hormone levels across groups. Group 7 had the highest serum TSH and T3 levels but the T4. In contrast, group 5, despite receiving the same EDs dose, exhibited lower TSH and T3 levels. Higher doses (20 mL/kg) corresponded to increased thyroid hormone alterations compared to lower doses.

**Table 8:** Mean values and standard deviation of T3, T4 and TSH of normal albino rats.

Groups	T3 (ng/dl)	T4 (μg/dL)	TSH (milli-international units/liter)
Control group (G1)	60.03 ± 1.88^f^	5.22 ± 0.03^a^	2.35 ± 0.12^f^
10% ED1 (G2)	68.65 ± 2.91^d^	4.01 ± 0.02^c^	3.40 ± 0.02^d^
20% ED1 (G3)	81.22 ± 1.09^b^	2.53 ± 0.01^e^	5.19 ± 0.52^b^
10% ED2 (G4)	65.21 ± 1.34^e^	4.67 ± 0.03^b^	3.03 ± 0.21^e^
20% ED2 (G5)	76.01 ± 1.65^c^	3.19 ± 0.04^d^	4.82 ± 0.42^c^
10% ED3 (G6)	71.34 ± 2.93^d^	3.97 ± 0.02^c^	3.73 ± 0.31^d^
20% ED3 (G7)	86.08 ± 1.63^a^	1.46 ± 0.05^f^	5.69 ± 0.01^a^
LSD	4.05	0.42	0.34

**Notes.**

Values are mean ± SD. Values in the same column sharing the same superscript letters are not statistically significantly different at (*p* ≤ 0.05).

### Histopathological findings of the liver

**Figure 3:**
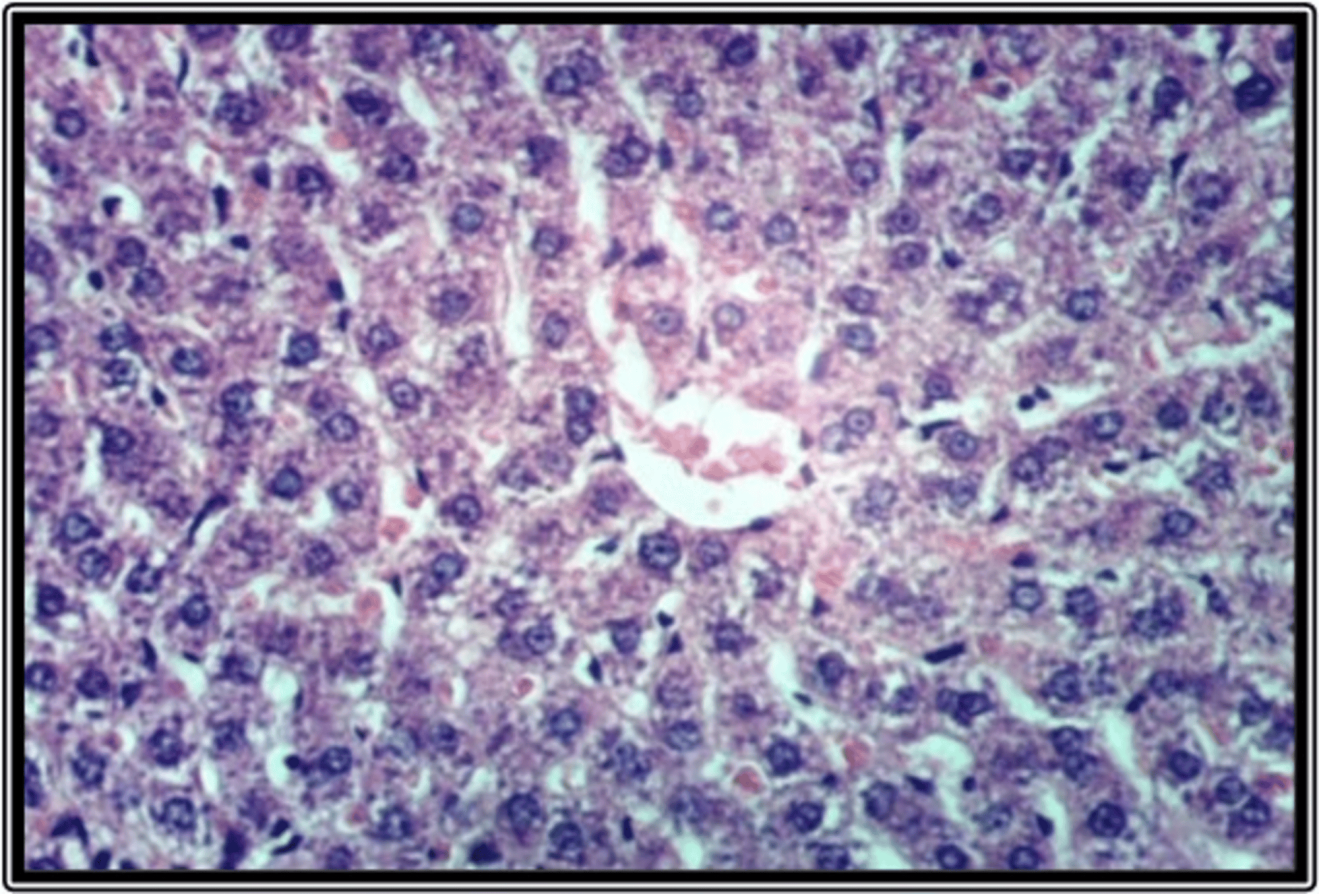
Liver of rat fed on basal diet as control group.

**Figure 4:**
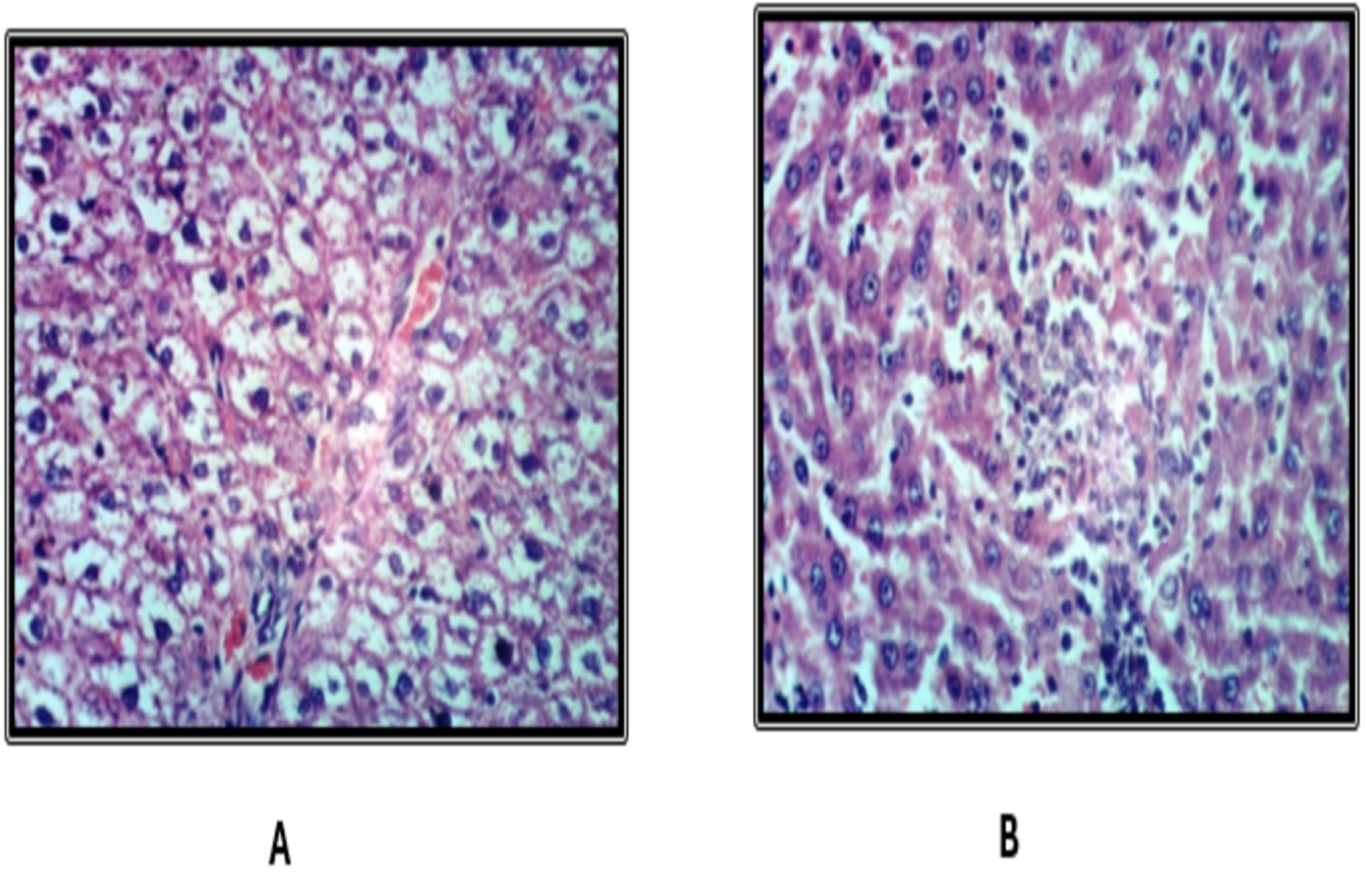
Liver of rat fed on basal diet and consuming 10 ml and 20 ml kgbw ED type1.

**Figure 5:**
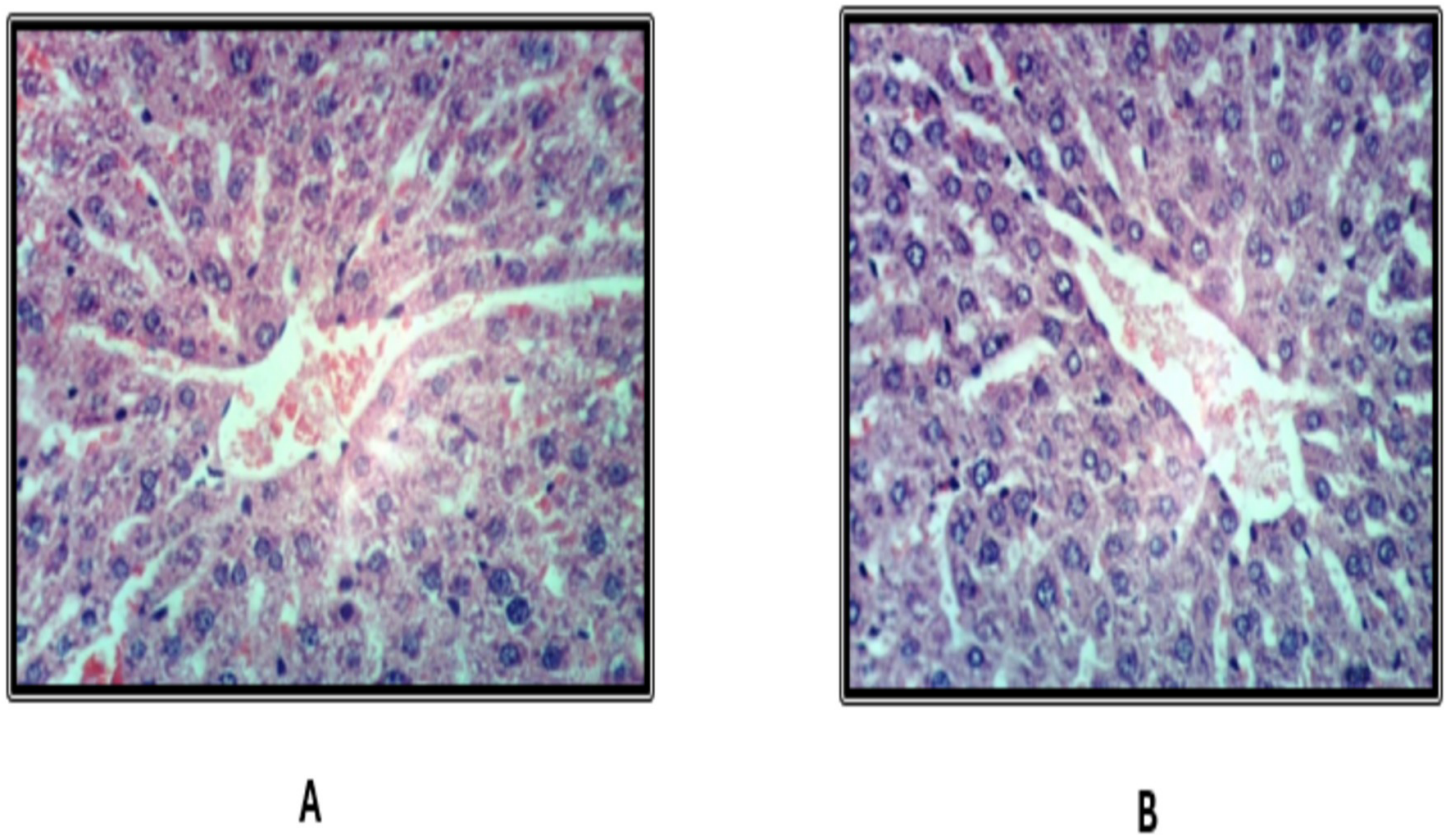
Liver of rat fed on basal diet and consuming 10 ml and 20 ml kgbw ED type 2.

**Figure 6:**
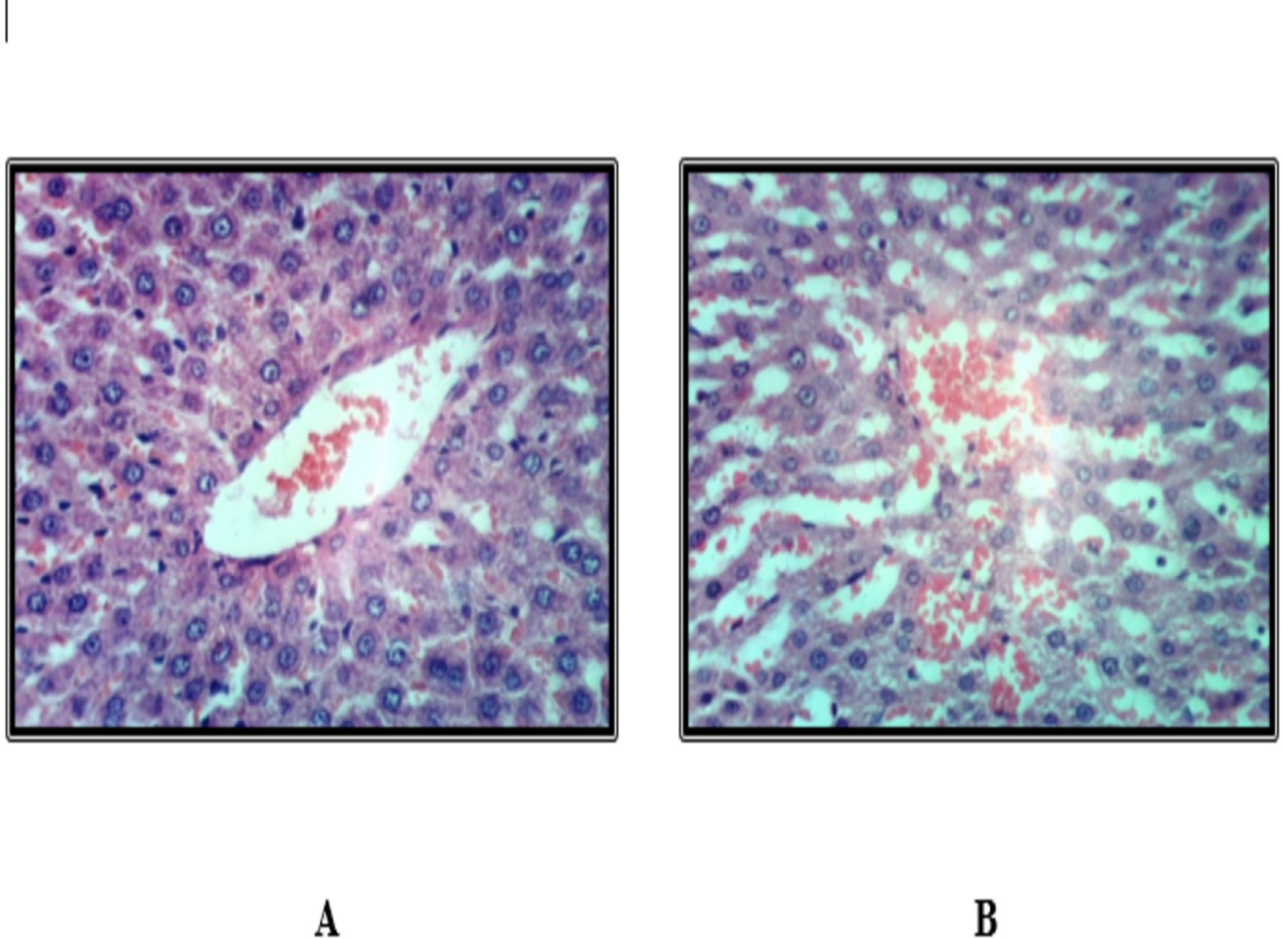
Liver of rat fed on basal diet and consuming 10 ml and 20 ml kgbw ED type 3.

Histological examination of liver tissues from the control group revealed a normal hepatic lobular architecture (Fig. 3). In contrast, Groups 2 and 5 exhibited hydropic degeneration of hepatocytes (Figs. 4A and 4B). Group 4 demonstrated mild vacuolization of hepatocytes (Fig. 5A), while Group 5 also presented additional signs of hepatocellular degeneration (Fig. 5B). Groups 3 and 6 showed activation of Kupffer cells and congestion of hepatic sinusoids, accompanied by infiltration of mononuclear inflammatory cells (Fig. 6A). Moreover, liver sections from Group 7 displayed marked activation of Kupffer cells and central veins, together with extensive mononuclear cell infiltration and localized areas of hepatic necrosis (Fig. 6B).

## Discussion

EDs are heavily marketed at events such as rock concerts and sporting competitions, often packaged attractively and promoted as enhancers of mental and physical performance. Approximately one-quarter of young adults report frequent consumption of EDs in amounts exceeding 300 mL per day (Trapp et al., 2014). However, these drinks have been associated with various adverse health effects, including Multiple Sclerosis (MS) largely due to their high calorie, sugar, and caffeine content. According to Graneri et al. (2021), MS is defined as a cluster of morphometric, physiological, and metabolic abnormalities that increase the risk of obesity, dyslipidemia, hyperglycemia, atherosclerotic CVD, and endothelial dysfunction.

Consequently, the objective of this investigation was to evaluate the potential effects of EDs on biochemical parameters and body weight gain in normal albino male rats over an eight-week period at doses of 10 and 20 mL/kg body weight. The results indicated that ED consumption significantly increased weight gain compared to the control group, with the greatest effect observed in rats administered the third type of ED, followed by the first type. Moreover, an increase in the ED dose corresponded to a significant rise in body weight. Specifically, body weight increased by 40.79%, 27.98%, and 21.36% over the control group in rats receiving 20 mL/kg body weight of ED groups 7, 3, and 5, respectively. This was supported by adiposity index calculations, which showed values of 0.88, 0.56, and 0.52 for these groups, compared to 0.34 in the normal control group, reflecting increased fat accumulation.

These findings align with previous studies in rats, which attributed significant weight gain to enhanced catabolism driven by elevated insulin levels induced by the sweetening agents in EDs, subsequently increasing lipid storage in adipose tissue (Malik et al., 2010). Similarly, Adjene, Emojevwe & Idiapho (2014) reported that sugars such as sucrose, glucose, and high-fructose corn syrup are the primary sweetening agents in EDs, and their excessive consumption has been strongly implicated in the global rise of obesity and related metabolic disorders. These sugars exert their metabolic effects through the activation of sweet taste receptors coupled to Gαs proteins (GPCRαs), which stimulate intracellular cAMP pathways. Such signaling may contribute to altered glucose homeostasis, lipid accumulation, and ultimately the development of MS, consistent with the findings observed in the present study. The high sugar content in EDs delivers calories in liquid form, which has been associated with lower satiety compared to solid foods. This may lead to incomplete compensatory reduction in subsequent energy intake, potentially contributing to a positive energy balance, as reported in previous studies (Malik & Hu, 2019). Additionally, the high glycemic load of these beverages can induce rapid fluctuations in blood glucose and insulin, which may enhance appetite and promote overconsumption. These factors have been implicated in the development of overweight and obesity with habitual ED consumption. Consumption of a Western-style diet rich in saturated fatty acids further exerts detrimental effects on metabolic health through multiple pathophysiological mechanisms. Notably, sugar-free formulations of EDs, while reducing overall sugar intake, have also been reported to induce insulin resistance, dyslipidemia, and inflammatory responses comparable to those elicited by sugar-sweetened EDs (Graneri et al., 2021). Collectively, these findings underscore the potential metabolic risks associated with increasing consumption of both regular and sugar-free EDs.

Chronic consumption of the three EDs significantly altered the metabolic profile of rats. All treated groups exhibited elevated fasting blood glucose and leptin levels, accompanied by reduced insulin concentrations compared with the control group, indicating insulin resistance and leptin dysregulation key hallmarks of MS. Among the beverages, Black produced the most pronounced disturbances, showing the highest glucose and leptin levels and the lowest insulin concentrations. These effects were dose-dependent, with the higher dose (20 mL/kg body weight) producing greater metabolic disruptions.

Quantitatively, blood glucose levels increased by 67.36–102.98%, and leptin levels by 25.02–43.37%, whereas insulin levels decreased by 38.36–48.68% relative to controls. Such variations likely reflect differences in the formulations of the drinks, particularly in their caffeine, sugar, and taurine content. High sugar and caffeine levels are known to impair insulin sensitivity and promote hyperglycemia, while taurine may modulate leptin secretion and energy balance.

These findings are consistent with previous reports indicating that chronic EDs consumption promotes oxidative stress, hormonal imbalance, and metabolic dysregulation (Abdelwahab et al., 2020; Khayyat et al., 2014a). Similarly, Nowak, Gośliński & Nowatkowska (2018) observed increased blood glucose and reduced insulin levels following EDs intake, attributed to high sugar content, while Malik et al. (2010) reported that elevated sweetener levels can increase leptin secretion and suppress insulin release. Collectively, these results highlight the potential of chronic EDs consumption to disrupt glucose homeostasis and hormonal regulation in a dose- and formulation-dependent manner.

Furthermore, excessive caffeine intake from EDs may promote glycogenolysis in the liver and muscles, temporarily raising blood glucose levels. Abonar et al. (2022) further demonstrated that caffeine reduces insulin sensitivity, impairs glucose metabolism, and elevates stress hormone levels, leading to hyperglycemia, gluconeogenesis, and lipolysis. These metabolic disturbances may inhibit peripheral glucose uptake *via* suppression of glycolytic enzymes. The resulting hyperglycemic state can trigger glycation of membrane phospholipids and organelles, potentially causing lipid peroxidation and DNA damage. In contrast, González-Domínguez et al. (2017) observed that beverages sweetened with sugar and containing caffeine may cause a simultaneous rise in both glucose and insulin levels within 20–30 min post-consumption, due to the synergistic effects of sugar and caffeine.

The current trial demonstrated that the Alb/Glb ratio, total proteins, globulin, and Alb are all influenced by EDs to some degree. The mean values of these parameters were significantly reduced following the consumption of EDs, particularly in rats that were co-administered a high dose of energy drink (20 ml/kg bw). The reduction percentage ranged between 40.37–53.56, 57.14–70.82, and 24.95–60.53% for total protein, Alb and Glb, respectively. Similar findings to those reported in this study were reported by Worthley et al. (2010) who showed that EDs lower the concentrations of Alb and Tp. Another trial, carried out by Famurewa et al. (2015), was aligned with our findings regarding lowering Alb and in contrast with the present results in increasing total protein.

Additionally, renal involvement is indicated by the elevated plasma urea, uric acid, and creatinine levels in rats that were administered EDs in comparison to the control group. Urea and creatinine are both byproducts of protein metabolism, which accumulate in the bloodstream when the kidneys are impaired. The results of this trial were consistent with those of prior research. The administration of EDs to rats leads to a significant rise in blood urea and creatinine concentrations, as indicated by previous research. Compromised renal function is frequently associated with elevated blood concentrations of urea and creatinine (Khayyat et al., 2014b). Caffeine, a major constituent of EDs, acts as a nonselective antagonist of adenosine receptors, particularly the A1 and A2A subtypes. In the kidney, blockade of adenosine A1 receptors interferes with the normal vasodilatory and autoregulatory actions of adenosine on the afferent arteriole. This antagonism can lead to afferent arteriolar constriction, reduced renal blood flow, and a decrease in the glomerular filtration rate (GFR). Consequently, impaired renal hemodynamics may contribute to elevated blood urea nitrogen (BUN) and serum creatinine, both of which are clinical markers of reduced renal function. At the same time, caffeine promotes diuresis by inhibiting tubular sodium and water reabsorption, which explains the paradoxical coexistence of increased urine output and potential markers of renal impairment in excessive intake (Akande & Banjoko, 2011).

Our research demonstrated a strong relationship between the consumption of high doses of EDs and kidney function levels. These findings are consistent with the results of another study, which demonstrated that kidney function levels increased significantly (*P* < 0.05) in response to high doses of EDs. The liver’s significant role in the production of urea through the urea cycle is underscored by the fact that elevated urea concentrations are frequently indicative of advanced liver disease. Urea cycle defects, a rare group of conditions that are defined by an inherited disruption of any one of the five enzymes in the urea cycle, can result in an increase in urea synthesis and a corresponding increase in plasma/serum urea concentration (Jafar, 2024).

Furthermore, the presence of liver injury caused by toxic substances is indicated by increases in the blood concentrations of hepatic enzymes, which are considered reliable indicators. Rats have been observed to exhibit raised levels of serum AST, ALT, and ALP as a result of the consumption of energy beverages in large quantities (Graneri et al., 2021). The results of this trial are consistent with the findings of previous research undertaken by Khayyat et al. (2014b) and Mansy et al. (2017), which investigated the effect of energy drink consumption on plasma liver function outcomes. The latter trial observed a rise in liver enzymes, particularly the ALP enzyme. In contrast, the third group experienced an 84.56% increase in AST, while the ALP enzyme increased by 57.37% in comparison to the control group. However, a different trial discovered that rats who were given EDs and caffeine had lower AST levels, but there was no evidence of hepatic injury. It was also reported that EDs contain niacin, the only vitamin with a side effect profile that includes hepatotoxicity and has been associated with drug- or toxin-induced hepatocellular injury patterns on pathologic examination. Liver injury may result from the consumption of niacin at a rate as low as 300 mg per day (Abdelwahab et al., 2020).

The consumption of EDs has been associated with adverse health effects, particularly alterations in behavioral and metabolic parameters, which may contribute to increased cardiovascular risk. In the present study, ED administration was associated with significant changes in lipid metabolism, as reflected by elevated plasma TG,TC, and LDL-C, along with reduced HDL-C. These lipid profile alterations are recognized as risk factors for cardiovascular disease rather than direct indicators of cardiac pathology (Wassef, Kohansieh & Makaryus, 2017).

To further assess cardiovascular risk, the CRI was calculated as the ratio of total cholesterol to HDL-C (CRI = TC/HDL-C). This index reflects the balance between atherogenic and protective lipoproteins, with higher values indicating an increased risk of cardiovascular disease. Importantly, an elevated CRI represents a risk marker and does not constitute a diagnosis of cardiovascular or cardiac abnormalities (Graneri et al., 2021).

The observed dyslipidemia may be partially attributed to the high sugar and stimulant content of EDs. Excessive intake of sugars has been shown to adversely affect lipid metabolism and cardiometabolic health, while caffeine and other stimulants may acutely increase heart rate and blood pressure (Wassef, Kohansieh & Makaryus, 2017). However, as direct assessments of cardiac structure or electrical activity were not performed in this study, no conclusions can be drawn regarding the presence of cardiac abnormalities.

Furthermore, EDs often contain bioactive compounds such as caffeine, guarana, and ginseng, whose combined cardiovascular effects remain incompletely understood. Previous studies have suggested that excessive or chronic consumption of such ingredients may influence cardiac electrophysiology and increase the risk of arrhythmias, particularly in susceptible populations (Wassef, Kohansieh & Makaryus, 2017; Costa, Rocha & Santos, 2023). Nevertheless, these potential effects should be interpreted cautiously and warrant further investigation using direct cardiac assessments.

The present study’s observation of increased serum calcium alongside decreased BMC and BMD can be explained by the adverse effects of ED consumption on bone metabolism. High sugar levels from EDs negatively impact bone health by promoting urinary calcium loss and disrupting calcium homeostasis (Ahn & Park, 2021). Supporting this, Berman et al. (2022) found an inverse relationship between the overconsumption of sugar-sweetened beverages (SSBs) and bone health, including bone fractures, BMC, and BMD.

Beyond sugar, three critical factors influence bone metabolism: phosphate, acidity, and caffeine. High caffeine intake often correlates with low calcium consumption. Caffeine adversely affects calcium absorption and increases the risk of bone density loss, potentially leading to osteoporosis and fractures. Chen et al. (2021) demonstrated that caffeine disrupts bone metabolism by increasing urinary calcium excretion, reducing vitamin D receptor expression, and decreasing 1,25 (OH)_2_D_3_-induced ALP activity in osteocytes. Additionally, Berman et al. (2022) reported that caffeine negatively impacts BMD by modulating adenosine receptors, inhibiting bone formation, and accelerating bone resorption.

Thyroid hormones play a crucial role in systemic metabolism and neurological development. In this study, prolonged EDs consumption was associated with significantly elevated serum T3 and TSH levels, alongside decreased thyroxine (T4) levels. These alterations suggest thyroid dysfunction linked to metabolic disorders caused by chronic intake of high caffeine content, which affects pituitary hormone secretion *via* the hypothalamic-pituitary-adrenal (HPA) axis (Ahmed, 2019; Zheng et al., 2023).

Histopathological analysis of liver tissues revealed that the EDs, especially the third type, induced central vein congestion, activation of hepatic sinusoids and Kupffer cells, and local infiltration of mononuclear cells associated with hepatic necrosis. Other groups showed milder effects, such as slight Kupffer cell activation or mild hydropic degeneration of hepatocytes. These findings align with previous research indicating that caffeinated EDs have detrimental effects on hepatocytes (Akande & Banjoko, 2011). Ismail et al. (2023) further noted that the severity of EDs effects depends on the amount consumed. Similarly, Afify et al. (2019) attributed hepatic cytoplasmic vacuolations to lipid droplet accumulation linked to hepatocyte degeneration.

There are a few limitations to this study that should be taken into account. Because of some physiological and metabolic similarities to humans, the rat model is a well-established tool for studying MS; nonetheless, it does not adequately represent the complexity of human reactions to long-term energy drink usage. As a result, care should be taken when extrapolating results to human populations. Despite the use of randomisation and controlled housing settings, inter-individual heterogeneity in the metabolic responses to energy drink intake is one potential source of bias. Additionally, because sex-specific differences in metabolic outcomes cannot be assessed, the results’ generalisability is limited by the use of only male rats. Furthermore, even if standardised procedures were used to lower measurement errors, biochemical and physiological assays still have some degree of imprecision. The study’s statistical power may also be limited by the comparatively small sample size per group, which raises the possibility of Type II errors and lowers the sensitivity to identify minor effects. Also, in this study, cardiovascular outcomes were inferred indirectly, as no direct structural or functional cardiac assessments were performed. Cardiovascular risk was estimated using biochemical indicators, including the serum lipid profile and a calculated cardiovascular risk index, which serve as proxy measures rather than definitive evidence of cardiac pathology. Therefore, the findings reflect susceptibility to cardiovascular disorders and should be interpreted with caution. Future investigations should include direct cardiac evaluations, such as echocardiography, electrocardiography, histopathological assessment, or biomarkers of myocardial injury, to substantiate these findings.

## Conclusion

This study reveals that sustained intake of high doses of EDs leads to multiple physiological disturbances. Specifically, it impairs liver and kidney function, alters the normal regulation of key metabolic hormones, compromises skeletal integrity, and may influence cardiovascular risk. These disruptions are associated with an increased likelihood of developing a range of chronic conditions, including metabolic disorders such as obesity and type 2 diabetes, endocrine imbalances like hypothyroidism, reduced bone mineral density potentially leading to osteoporosis, and progressive hepatic and renal impairment. Given these health implications, it is imperative to limit ED consumption and approach their use with informed cautio.

## Supplemental Information

10.7717/peerj.20926/supp-1 Supplemental Information 1 Raw Data

10.7717/peerj.20926/supp-2 Supplemental Information 2 Author Checklist

## Additional Information and Declarations

### Data Availability

The following information was supplied regarding data availability: The raw data are available in the Supplemental File.

## References

[ref-1] Abdelwahab W, Elsayed S, Afify A, Mohammed A, Abd AlRahman R (2020). Study of the biochemical, histological and cytogenetic effects of two different energy drinks (EDs); Red bull and power horse; on brain of adult male Albino rats and to determine the possible protective role of omega-3 on the adverse effects of EDs. Journal of Recent Advances in Medicine.

[ref-2] Abonar M, Aboraya A, Elbakary N, Elwan W (2022). Effect of energy drink on the pancreas of adult male albino rat and the possible protective role of avocado oil: histological and immunohistochemical study. Egyptian Journal of Histology.

[ref-3] Abrahamsen B, Gram J, Hansen TB, Beck-Nielsen H (1995). Cross calibration of QDR-2000 and QDR-1000 dual-energy X-ray densitometers for bone mineral and soft-tissue measurements. Bone.

[ref-4] Acar P, Küme T, Gürsoy Ç, Demir BA (2018). Serum galectin-1 levels are positively correlated with body fat and negatively with fasting glucose in obese children. Peptides.

[ref-5] Adjene J, Emojevwe V, Idiapho D (2014). Effects of long-term consumption of energy drinks on the body and brain weights of adult Wistar rats. Journal of Experimental and Clinical Anatomy.

[ref-6] Afify AM, Abd Elwhab WA, Mohammed AA, Al-Rahman A, Rehab A, El-Sayed SB (2019). Study of the protective role of omega 3 on hepatotoxicity induced by different energy drinks in adult male albino rats. Zagazig Journal of Forensic Medicine.

[ref-7] Ahmed RG (2019). Gestational caffeine exposure acts as a fetal thyroid-cytokine disruptor by activating caspase-3/BAX/Bcl-2/Cox2/NF-*κ*B at ED 20. Toxicological Research.

[ref-8] Ahn H, Park YK (2021). Sugar-sweetened beverage consumption and bone health: a systematic review and meta-analysis. Nutrition Journal.

[ref-9] Akande IS, Banjoko OA (2011). Assessment of biochemical effect of “Power Horse” energy drink on hepatic, renal and histological functions in sprague dawley rats. Annual Review & Research in Biology.

[ref-10] Ariffin H, Chong XQ, Chong PN, Okechukwu PN (2022). Is the consumption of energy drink beneficial or detrimental to health: a comprehensive review?. Bulletin of the National Research Centre.

[ref-11] Bancroft JD, Gamble M (2008). Theory and practice of histological techniques.

[ref-12] Bedi N, Dewan P, Gupta P (2014). Energy drinks: potions of illusion. Indian Pediatrics.

[ref-13] Berman NK, Honig S, Cronstein BN, Pillinger MH (2022). The effects of caffeine on bone mineral density and fracture risk. Osteoporosis International.

[ref-14] Bhardwaj S, Bhattacharjee J, Bhatnagar MK, Tyagi S, Delhi N (2013). Atherogenic index of plasma. Castelli risk index and atherogenic coefficient-new parameters in assessing cardiovascular risk. International Journal of Pharmaceutical and Biological Sciences.

[ref-15] Breda JJ, Whiting SH, Encarnação R, Norberg S, Jones R, Reinap M, Jewell J (2014). Energy drink consumption in Europe: a review of the risks, adverse health effects, and policy options to respond. Frontiers in Public Health.

[ref-16] Busher JT (1990). Serum albumin and globulin. Clinical Methods: the History, Physical, and Laboratory Examinations.

[ref-17] Campbell JA (1963). Methodology of protein evaluation. RAG Nutrition Document R.

[ref-18] Chapman DG, Castillo R, Campbell JA (1959). Evaluation of protein in foods: 1. A method for the determination of protein efficiency ratios. Canadian Journal of Biochemistry and Physiology.

[ref-19] Chen Q, Kord-Varkaneh H, Santos HO, Genario R, Dang M (2021). Higher intakes of dietary caffeine are associated with 25-hydroxyvitamin D deficiency. International Journal for Vitamin and Nutrition Research.

[ref-20] Costa R, Rocha C, Santos H (2023). Cardiovascular and cerebrovascular response to RedBull^®^ energy drink intake in young adults. Anatolian Journal of Cardiology.

[ref-21] Costantino A, Maiese A, Lazzari J, Casula C, Turillazzi E, Frati P, Fineschi V (2023). The dark side of energy drinks: a comprehensive review of their impact on the human body. Nutrients.

[ref-22] Dobiasova M (2004). Atherogenic index of plasma [log (triglycerides/HDL-cholesterol)]: theoretical and practical implications. Clinical Chemistry.

[ref-23] Drury RA, Wallington EA (1980). Carleton’s histological technique.

[ref-24] El-Wakf AM, Hassan HA, Elsaid FG (2009). Hypothyroidism in male rats of different ages exposed to nitrate polluted drinking water. Research Journal of Medicine and Medical Sciences.

[ref-25] Ettinger SJ, Feldman EC (2005). Textbook of veterinary internal medicine.

[ref-26] Famurewa A, Folawiyo AM, Epete MA, Onuoha M, Igwe E (2015). Consumption of caffeinated energy drink induces alterations in lipid profile and hepatic aminotransferases in experimental rats. Journal of Chemical and Pharmaceutical Research.

[ref-27] Francisqueti FV, Nascimento AF, Minatel IO, Dias MC, Luvizotto RDAM, Berchieri-Ronchi C, Ferreira ALA, Corrêa CR (2017). Metabolic syndrome and inflammation in adipose tissue occur at different times in animals submitted to a high-sugar/fat diet. Journal of Nutritional Science.

[ref-28] Frank EA, Shubha MC, D’Souza CJ (2012). Blood glucose determination: plasma or serum?. Journal of Clinical Laboratory Analysis.

[ref-29] González-Domínguez R, Mateos RM, Lechuga-Sancho AM, González-Cortés JJ, Corrales-Cuevas M, Rojas-Cots JA, Segundo C, Schwarz M (2017). Synergic effects of sugar and caffeine on insulin-mediated metabolomic alterations after an acute consumption of soft drinks. Electrophoresis.

[ref-30] Graneri LT, Mamo JC, D’Alonzo Z, Lam V, Takechi R (2021). Chronic intake of energy drinks and their sugar free substitution similarly promotes metabolic syndrome. Nutrients.

[ref-31] Hamooya BM, Siame L, Muchaili L, Masenga SK, Kirabo A (2025). Metabolic syndrome: epidemiology, mechanisms, and current therapeutic approaches. Frontiers in Nutrition.

[ref-32] Hegsted D, Mills R, Perkins E (1941). Salt mixture. Journal of Biological Chemistry.

[ref-33] Henning Y, Vole C, Begall S, Bens M, Broecker-Preuss M, Sahm A, Szafranski K, Burda H, Dammann P (2014). Unusual ratio between free thyroxine and free triiodothyronine in a long-lived mole-rat species with bimodal ageing. PLOS ONE.

[ref-34] Hitz MF, Jensen JE, Eskildsen PC (2007). Bone mineral density and bone markers in patients with a recent low-energy fracture: effect of 1 year of treatment with calcium and vitamin D. American Journal of Clinical Nutrition.

[ref-35] Huang S, Czech MP (2013). Measurement of serum insulin levels by enzyme-linked immunosorbent assay. Methods in Molecular Biology.

[ref-36] Ismail NF, Hamdy G, Hassan AA, Elmetwalli A, Salah M, Hassan J (2023). The impact of energy drinks on liver health. Medical Journal of Viral Hepatitis.

[ref-37] Jafar SN (2024). Changes in biochemical and sperm parameters of rats drinking energy drinks. Cellular and Molecular Biology.

[ref-38] Kaiyu P, Rongliang T, Xiaocong Y, Zhongxin Z (2021). Associations between serum calcium, 25(OH)D level and bone mineral density in adolescents. Advances in Rheumatology.

[ref-39] Kaneko JJ, Harvey JW, Bruss ML (2008). Clinical biochemistry of domestic animals.

[ref-40] Kassab AA, Tawfik SM (2018). Effect of a caffeinated energy drink and its withdrawal on the submandibular salivary gland of adult male albino rats: a histological and immunohistochemical study. Egyptian Journal of Histology.

[ref-41] Khayyat LI, Essawy AE, Al Rawy MM, Sorour JM (2014a). Comparative study on the effect of energy drinks on haematopoietic system in Wistar albino rats. Journal of Environmental Biology.

[ref-42] Khayyat L, Essawy A, Sorour J, Al Rawi M (2014b). Impact of some energy drinks on the structure and function of the kidney in wistar albino rats. Life Science Journal.

[ref-43] Kikuchi Hayakawa H, Onodera N, Matsubara S, Yasuda E, Chonan O, Takahashi R, Ishikawa F (1998). Effects of soy milk and bifidobacterium fermented soy milk on lipid metabolism in aged ovariectomized rats. Bioscience, Biotechnology, and Biochemistry.

[ref-44] Liu J, Mao C, Mao X, Wang X, Zheng T, Dong L, Mao Y (2025). T3 and T4 autoantibodies: emerging biomarkers for evaluating thyroid disorders. Frontiers in Endocrinology.

[ref-45] Malik VS, Hu FB (2019). Sugar-sweetened beverages and cardiometabolic health: an update of the evidence. Nutrients.

[ref-46] Malik VS, Popkin BM, Bray GA, Després JP, Willett WC, Hu FB (2010). Sugar-sweetened beverages and risk of metabolic syndrome and type 2 diabetes: a meta-analysis. Diabetes Care.

[ref-47] Mansy W, Alogaiel DM, Hanafi M, Zakaria E (2017). Effects of chronic consumption of energy drinks on liver and kidney of experimental rats. Tropical Journal of Pharmaceutical Research.

[ref-48] Marinoni M, Parpinel M, Gasparini A, Ferraroni M, Edefonti V (2022). Risky behaviors, substance use, and other lifestyle correlates of energy drink consumption in children and adolescents: a systematic review. European Journal of Pediatrics.

[ref-49] Matsushima-Nagata K, Sugiuchi H, Anraku K, Takao T, Kondo Y, Ishitsuka Y, Kayahara N (2021). A homogeneous assay to determine high-density lipoprotein subclass cholesterol in serum. Analytical Biochemistry.

[ref-50] Miller M, Stone NJ, Ballantyne C, Bittner V, Criqui MH, Ginsberg HN, Goldberg AC, Howard WJ, Jacobson MS, Kris-Etherton PM, Lennie TA, Levi M, Mazzone T, Pennathur S (2011). Triglycerides and cardiovascular disease: a scientific statement from the American Heart Association. Circulation.

[ref-51] Nadeem IM, Shanmugaraj A, Sakha S, Horner NS, Ayeni OR, Khan M (2021). Energy drinks and their adverse health effects: a systematic review and meta-analysis. Sports Health.

[ref-52] Nowak D, Gośliński M, Nowatkowska K (2018). The effect of acute consumption of energy drinks on blood pressure, heart rate and blood glucose in the group of young adults. International Journal of Environmental Research and Public Health.

[ref-53] Reeves PG, Nielsen FH, Fahey Jr GC (1993). AIN-93 purified diets for laboratory rodents: final report of the American Institute of Nutrition *ad hoc* writing committee on the reformulation of the AIN-76A rodent diet. The Journal of Nutrition.

[ref-54] Rifai N (2017). Tietz textbook of clinical chemistry and molecular diagnostics (E-book).

[ref-55] Robinet P, Wang Z, Hazen SL, Smith JD (2010). A simple and sensitive enzymatic method for cholesterol quantification in macrophages and foam cells. Journal of Lipid Research.

[ref-56] Saad DE, Mansour SZ, Kandil EI, Hassan A, Moawed FS, Elbakry MM (2025). Boswellic acid synergizes with low-dose ionizing radiation to mitigate thioacetamide-induced hepatic encephalopathy in rats. BMC Pharmacology and Toxicology.

[ref-57] Stanhope KL (2016). Sugar consumption, metabolic disease and obesity: the state of the controversy. Critical Reviews in Clinical Laboratory Sciences.

[ref-58] Strubbe JH, Bruggink JE, Steffens AB (1998). Hepatic portal vein cannulation for infusion and blood sampling in freely moving rats. Physiology & Behavior.

[ref-59] Tietz NW (1995). Clinical guide to laboratory tests. Clinical guide to laboratory tests.

[ref-60] Trapp GS, Allen KL, O’Sullivan T, Robinson M, Jacoby P, Oddy WH (2014). Energy drink consumption among young Australian adults: associations with alcohol and illicit drug use. Drug and Alcohol Dependence.

[ref-61] Underwood W, Anthony R (2020). AVMA guidelines for the euthanasia of animals: 2020 edition.

[ref-62] Van Asselt E, Choudhary M, Clavica F, Van Mastrigt R (2017). Urethane anesthesia in acute lower urinary tract studies in the male rat. Laboratory Animals.

[ref-63] Wassef B, Kohansieh M, Makaryus AN (2017). Effects of energy drinks on the cardiovascular system. World Journal of Cardiology.

[ref-64] Worthley MI, Prabhu A, De Sciscio P, Schultz C, Sanders P, Willoughby SR (2010). Detrimental effects of energy drink consumption on platelet and endothelial function. The American Journal of Medicine.

[ref-65] Young DS, Friedman RB (2001). Effects of disease on clinical laboratory tests.

[ref-66] Zheng J, Zhu X, Xu G, Wang X, Cao M, Zhu S, Zhou Y (2023). Relationship between caffeine intake and thyroid function: results from NHANES 2007–2012. Nutrition Journal.

